# Static and dynamic functional connectivity variability of the anterior-posterior hippocampus with subjective cognitive decline

**DOI:** 10.1186/s13195-022-01066-9

**Published:** 2022-09-03

**Authors:** Qiang Wang, Ben Chen, Xiaomei Zhong, Le Hou, Min Zhang, Mingfeng Yang, Zhangying Wu, Xinru Chen, Naikeng Mai, Huarong Zhou, Gaohong Lin, Si Zhang, Yuping Ning

**Affiliations:** 1 Center for Geriatric Neuroscience, The Affiliated Brain Hospital of Guangzhou Medical University, Guangzhou, Guangdong Province China; 2 Department of Geriatric Psychiatry, The Second People’s Hospital of Dali Bai Autonomous Prefecture, Dali, Yunnan Province China; 3 Department of Neurology, The Affiliated Brain Hospital of Guangzhou Medical University, Guangzhou, Guangdong Province China; 4 The first School of Clinical Medicine, Southern Medical University, Guangzhou, Guangdong Province China; 5 Guangdong Engineering Technology Research Center for Translational Medicine of Mental Disorders, Guangzhou, China

**Keywords:** Alzheimer’s disease, Dementia, Subjective cognitive decline, Magnetic resonance imaging, Functional connectivity

## Abstract

**Background:**

Subjective cognitive decline (SCD) is a putative Alzheimer’s disease (AD) precursor without objective neuropsychological deficits. The hippocampus plays an important role in cognitive function and emotional responses and is generally aberrant in SCD. However, previous studies have mainly focused on static functional connectivity (sFC) by resting-state functional magnetic resonance imaging (fMRI) in SCD individuals, and it remains unclear whether hippocampal dynamic functional connectivity (dFC) changes exist in SCD and whether those changes are associated with subtle changes in cognitive function or affect.

**Methods:**

Seventy SCD patients and 65 healthy controls were recruited. Demographic data, comprehensive neuropsychology assessments, and resting-state fMRI data were collected. The bilateral anterior and posterior hippocampi were selected as seeds to investigate the static and dynamic functional connectivity alterations in SCD.

**Results:**

Compared to healthy controls, subjects with SCD exhibited: (1) decreased sFC between the left caudal hippocampus and left precuneus; (2) decreased dFC variability between the bilateral caudal hippocampus and precuneus; (3) increased dFC variability between the bilateral rostral hippocampus and caudate nucleus; and (4) increased dFC variability between the left rostral hippocampus and left olfactory cortex. Additionally, the attention scores were positively correlated with dFC variability between the left posterior hippocampus and left precuneus, and the dFC variability between the bilateral anterior hippocampus and caudate nucleus was positively correlated with depression scores and negatively correlated with global cognition scores.

**Conclusion:**

SCD individuals exhibited abnormal sFC and dFC in the anterior-posterior hippocampus, and abnormal dFC was more widespread than abnormal sFC. A combination of sFC and dFC provides a new perspective for exploring the brain pathophysiological mechanisms in SCD and offers potential neuroimaging biomarkers for the early diagnosis and intervention of AD.

**Supplementary Information:**

The online version contains supplementary material available at 10.1186/s13195-022-01066-9.

## Introduction

Subjective cognitive decline (SCD) is an individual’s self-report of cognitive decline, without abnormality of objective neuropsychological assessment [1]. SCD has been described as the earliest at-risk state of Alzheimer’s disease (AD), and it increases the risk for developing mild cognitive impairment (MCI) and future AD [2]. Studies have increasingly indicated that SCD is associated with specific and distinctive underlying AD pathological events, such as abnormal amyloid-β (Aβ) load [3] and tau deposition [4], reduced temporal cortical thickness [5] and hippocampal volume [6], disruptions in white matter [7], reduced glucose metabolism [8], and brain functional abnormalities [9].

Resting-state functional magnetic resonance imaging (rs-fMRI) provides a novel approach for reflecting internal functional connectivity (FC) by measuring blood oxygen level-dependent (BOLD) signals [10]. For SCD individuals, rs-fMRI studies have shown decreased static functional connectivity (sFC) strength in the left medial superior frontal, left precuneus, left parietal, right cuneus, and bilateral calcarine [11]; decreased sFC in the posterior memory system; decreased sFC between the retrosplenial cortex and precuneus [12]; increased sFC between the retrosplenial cortex and posterior cingulate cortex [13]; and increased occipital and parietal sFC associated with the severity of memory concerns [14]. Moreover, the sFC was decoupled between the hippocampus and posterior default mode network (DMN) but not between the hippocampus and anterior DMN with SCD [15]. Therefore, altered sFC can serve as an effective and noninvasive approach for exploring the neural mechanisms underlying preclinical at-risk AD patients.

Currently, most of the above rs-fMRI studies have focused on sFC, and the dynamic characteristics of brain function in SCD subjects have not been fully investigated. Accumulating studies have suggested that the brain is intrinsically a dynamic system with discrete model switching rapidly [16], and dynamic indices based on sliding-window changes may be more informative than static indices [17]. Recently, studies have shown that SCD exhibits significantly increased fractional windows and mean dwell time [18] and decreased occurrence frequency [19] of a DMN-dominated dynamic functional connectivity (dFC) state compared to that of healthy controls. The significant role of dFC in SCD has been gradually recognized, but previous studies were based on whole-brain network-based analyses, and little research has been performed using seed-to-voxel-based analyses.

The hippocampus plays a critical role in cognitive function and emotional responses [20], and it is affected very early during AD pathogenesis [21]. Additionally, the extent of hippocampal neurofibrillary involvement is strongly correlated with AD symptoms and disease course [22]. The diverse functions of the hippocampus are partially explained by functional differences along its longitudinal axis. The dominant view is that the posterior (or dorsal) hippocampus is implicated in memory and spatial navigation and that the anterior (or ventral) hippocampus mediates affective-related behaviors [23]. Recent conjunction analysis also showed a reduction in anterior-posterior hippocampal functional network convergence strength from early MCI to AD [24]. Although SCD is generally believed to represent subtle changes in cognitive function [25] and subclinical mood disorders [26], it remains unclear whether anterior-posterior hippocampal dynamic functional connectivity changes exist in SCD and whether those changes are associated with subtle changes in cognitive function or affect.

Therefore, the present study aimed to explore the static and dynamic FC of the anterior-posterior hippocampus in SCD individuals using the sliding-window method. We hypothesized that (1) altered anterior-posterior hippocampal dynamic functional connectivity is already present with SCD, and abnormal dFC is more widespread than abnormal sFC; (2) dFC in the posterior hippocampal system is associated with subtle changes in cognitive function, and dFC in the anterior hippocampal system is associated with subtle changes in affect.

## Materials and methods

### Subjects

The current research included 70 participants with SCD matched for age, sex, and years of education with 65 healthy controls (HCs). All participants were recruited from the Affiliated Brain Hospital of Guangzhou Medical University and the community in Guangzhou. All participants or their legal guardians provided signed informed consent to participate in the study. The study was conducted according to the Declaration of Helsinki and approved by the ethics committees of the Affiliated Brain Hospital of Guangzhou Medical University.

The SCD criteria included the following two major features [1]: a self-experienced persistent decline in cognitive capacity relative to a previously normal cognitive status unrelated to an acute event and a normal performance on standardized cognitive tests used to classify MCI, adjusted for age, sex, and years of education. The diagnostic criteria of MCI were based on the Peterson criteria [27]. HC individuals were age-matched, cognitively healthy individuals without memory complaints. All recruited subjects with a Hachinski score higher than 4 were excluded [28]. Individuals with a history of stroke, neuropsychiatric disorders (Parkinson’s disease, epilepsy, brain tumor, etc.), severe anxiety or depression, and other psychiatric disorders (such as schizophrenia, bipolar disorder, posttraumatic stress disorders, and panic disorder) were excluded. All subjects underwent structured interviews, clinical symptoms, and comprehensive cognitive assessments.

### Neuropsychological assessments

The standardized cognitive evaluation was performed by an experienced psychologist. Global cognitive function was measured using the Mini-Mental State Examination (MMSE) [29] and Memory and Executive Screening (MES) [30], which is a valid and easily administered cognitive screening tool with high sensitivity and specificity for global cognition in Chinese. Its score ranges from 0 to 100, with a higher score indicating better cognition. The five cognitive domains were evaluated by the following neuropsychological tests: (1) Auditory Verbal Learning Test delay recall (AVLT-DR) [31], (2) executive function tested with the time of Part B of the Trail-Making Test (TMTB) [32], (3) language function evaluated with the Animal Verbal Fluency Test (AVFT) [33], (4) attention function tested with the Symbol Digit Modalities Test (SDMT) [34], and (5) visuospatial skill assessed with the Rey-Osterrieth Complex Figure Test (ROCF) [35]. Depressive symptoms were measured using the Geriatric Depression Scale (GDS) [36]. The scores for the cognitive domains and depressive symptoms were calculated by transforming each of the tests into standardized *z* scores.

### Image acquisition

Imaging data were acquired by the Philips 3.0 T MR systems in The Affiliated Brain Hospital of Guangzhou Medical University (Philips, Achieva, Netherlands). Sagittal resting-state fMRI datasets of the whole brain were acquired in 8 minutes using a single-shot gradient echo-planar imaging (EPI) pulse sequence with the following parameters: TE = 30 ms, TR = 2000 ms, flip angle (FA) = 90°, number of slices = 33, slice thickness = 4 mm, matrix size = 64 × 64, and field of view (FOV) = 220 × 220 mm.

### Image preprocessing

Preprocessing for rs-fMRI data was performed using the data processing assistant for rs-fMRI advanced edition (DPARSF, vision 5.1, http://rfmri.org) (RRID:SCR_010501) [37], which is based on Statistical Parametric Mapping (SPM12, http://www.fil.ion.ucl.ac.uk/spm/) (RRID:SCR_007037). The first ten volumes were discarded to preserve steady-state data. The 230 remaining images were corrected for timing differences and head motion. A record of the head motion was provided after realignment correction. Subjects who had images with more than 2 mm translational movement or more than 2° rotational movement were excluded from further analysis. Then, the motion-corrected images were spatially normalized into a standard Montreal Neurological Institute (MNI) (RRID:SCR_000021) echo planar imaging (EPI) template, resliced to a voxel size of 3 × 3 × 3 mm^3^ resolution and smoothed using a 4 mm full width at half maximum (FWHM) Gaussian kernel, and detrending was then carried out. Linear trend and nuisance covariates were then regressed out from each time series, including signals of white matter and cerebrospinal fluid as well as the Friston-24 parameters of head motion [38]. Finally, a bandpass filter (0.01 Hz<f<0.1 Hz) was applied to reduce the effect of low-frequency drifts and high-frequency noise [39].

### Definition of regions of interest

The bilateral anterior/posterior hippocampus was defined as regions of interest (ROIs) according to the Brainnetome Atlas (Brainnetome Atlas Viewer, vision 1.0, http://atlas.brainnetome.org/) (RRID:SCR_014091) [40]. The Brainnetome atlas comprises 246 cortico-subcortical grey matter regions based on the structural and functional connectional architecture of the human brain and allows for annotation of behavioral domains [40].

### Estimation of static and dynamic functional connectivity

Pearson’s correlation coefficients were determined between the time courses of all voxels within each ROI and the time courses of each voxel in the whole brain, which is defined as sFC [10] and reflects brain static connectivity patterns. The dFC variability patterns were characterized using the sliding-window approach, which sliced ROI time courses into several short data segments with 50 TR window lengths and step widths of 1 TR for each segment on the Temporal Dynamic Analysis (TDA) toolkits integrated in DPABI software (http://rfmri.org/DPABI) (RRID:SCR_010501). In total, 181 sliding windows of dFC were obtained. For each sliding window, correlation maps were produced by computing the temporal correlation coefficient between the truncated time series of the bilateral anterior/posterior hippocampus seeds and all the other voxels. Consequently, 181 sliding-window correlation maps were obtained for each individual. To improve the normality of the correlation distribution, each correlation map was converted into *z* value maps using Fisher’s *r*-to-*z* transformation. Then, the dFC maps were computed by calculating the standard deviation of 181 sliding-window *z* value maps. Then, *z*-standardization was applied for the dFC maps. Finally, all the dFC maps were smoothed using a 4-mm full width at half maximum Gaussian kernel [41]. In addition, to exclude the influence of window width, smaller window sizes of 30 TRs were tested, and the results were very consistent with the results of 50 TRs (see supplementary materials 1). Meanwhile, results obtained without smoothing are available in the Supplementary material 2.

### Statistical analyses

Independent-sample *t* tests and two-tailed chi-square tests were used to compare demographic data and neuropsychological scores between the two groups using Statistical Package for Social Sciences version 25.0 (IBM SPSS 25.0, Chicago, IL, USA) (SCR_002865).

The mean time series of the left and right anterior/posterior hippocampus were extracted. A voxelwise dFC analysis was performed by computing the temporal cross-correlation between the mean time series of each ROI and the time series of each voxel within the brain. The correlation coefficients of each voxel were normalized to *Z* scores with Fisher’s *r*-to-*z* transformation. Therefore, an entire brain *Z* score map was created for each ROI of each subject.

The one-sample *t* test was performed on z score maps for each ROI to demonstrate the within-group dFC and sFC spatial distribution of each seed for the patients in the SCD and HC groups, and the significance level was set at *p* < 0.05 (uncorrected). Then, a two-sample *t* test was performed to assess the significant differences in whole-brain dFC and sFC in each region between SCD patients and HCs within the union mask of the one-sample *t* test results of both groups. The control variables included age, sex, and years of education. Gaussian random field (GRF) theory was used for cluster-level multiple comparison correction (voxel *p* value < 0.001; cluster *p* value < 0.05). The mean *z* values were extracted when statistically significant group differences were observed in dFC and sFC. Then, partial correlation analysis was used to compute the correlation between neuroimaging indicators and neuropsychological scores. Age, sex, and years of education were included as nuisance covariates in all correlation analyses.

## Results

### Demographic and neuropsychological information

The demographic and neuropsychological information of different subjects is listed in Table 1. No differences in age, sex, or years of education were observed between the SCD and HC groups (*p* > 0.05). For cognitive and depressive performance, there was no significant difference between the SCD and HC groups (*p* > 0.05).

**Table 1:** Demographic data, clinical information, and neurophysiological performance of SCD and HC

	HC (*n* = 65)	SCD (*n* = 70)	*t*/*χ*^2#^	*p*
Male (%)	22 (33.8%)	23 (32.9%)	0.02	0.90
Age	65.9 ± 5.1	67.0 ± 5.6	− 1.09	0.28
Years of education	11.0 ± 3.0	11.5 ± 2.9	− 1.12	0.27
**Global cognition**				
MMSE	27.3 ± 2.0	27.3 ± 1.9	− 0.02	0.98
MES	87.7 ± 7.4	84.3 ± 14.4	1.71	0.09
**Memory**				
AVLT-DR	6.7 ± 2.0	6.7 ± 2.6	0.27	0.98
**Language**				
AVFT	14.8 ± 3.6	15.7 ± 3.8	− 1.48	0.14
**Executive function**				
TMTB	60.1 ± 20.2	59.6 ± 19.6	1.47	0.88
**Visuospatial skill**				
ROCF	27.7 ± 3.6	27.8 ± 3.7	− 0.18	0.86
**Attention**				
SDMT	34.9 ± 10.3	36.3 ± 9.7	− 0.77	0.45
**Depression**				
GDS	2.0 ± 1.7	2.1 ± 1.9	− 0.36	0.72

Means ± standard deviation

*Abbreviations*: *HC* healthy controls, *SCD* subjective cognitive decline, *MMSE* Mini-Mental State Examination, *MES* Memory and Executive Screening, *AVLT-DR* Auditory Verbal Learning Test Long-term delayed recall, *AVFT* Animal Verbal Fluency Test, *TMTB* Part B of Trail-Making Test, *ROCF* Rey-Osterrieth Complex, *SDMT* Symbol-Digit Modality Test, *GDS* Geriatric Depression Scale

^#^*t* refers to the independent samples test, and *χ*^2^ refers to the 2-tailed chi-square test

### Comparison of functional connectivity in the region of interest

**Fig. 1:**
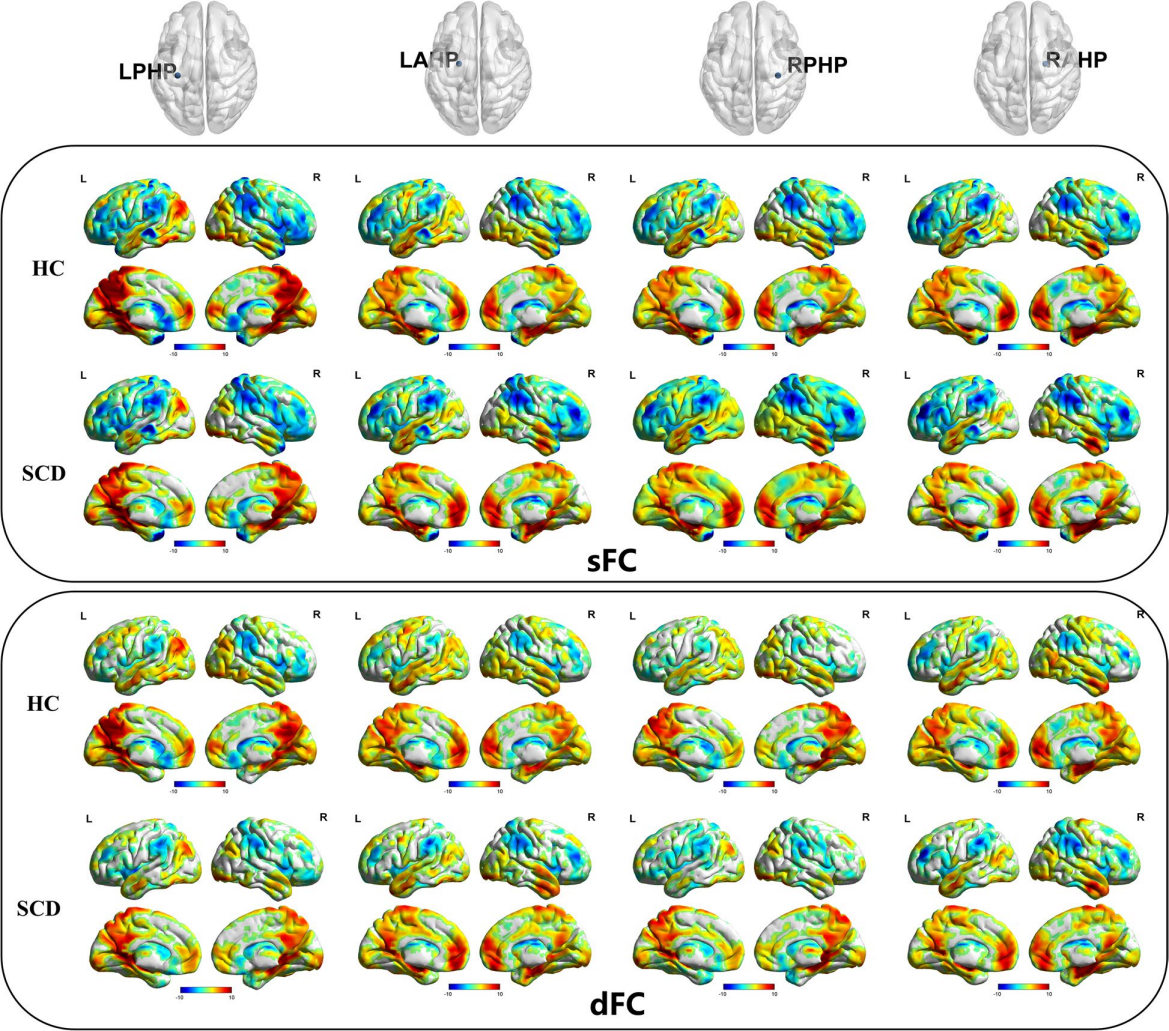
The one-sample *t* test of functional connectivity patterns of the bilateral anterior and posterior hippocampus in the SCD and HC groups. The one-sample *t* test of sFC and dFC showed that the bilateral posterior hippocampus and anterior hippocampus with high sFC and dFC values were mainly connected to the bilateral frontal cortex, temporal cortex, and parietal cortex. The bilateral anterior hippocampus with high dFC and sFC values was mainly connected to the bilateral frontal cortex, temporal lobe, occipital cortex, parietal cortex and limbic cortex. LAHP, left anterior hippocampus; LPHP, left posterior hippocampus; RAHP, right anterior hippocampus; RPHP, right posterior hippocampus; HC, healthy controls; SCD, subjective cognitive decline; sFC, static functional connectivity; dFC, dynamic functional connectivity

The one-sample *t* test of sFC and dFC showed that the bilateral posterior hippocampus and anterior hippocampus with high sFC and dFC values were mainly connected to the bilateral frontal cortex, temporal cortex, and parietal cortex. The bilateral anterior hippocampus with high dFC and sFC values was mainly connected to the bilateral frontal cortex, temporal lobe, occipital cortex, parietal cortex, and limbic cortex (Fig. 1). The spatial distributions of dFC and sFC values in the SCD group were similar to those in the HC group.

**Fig. 2:**
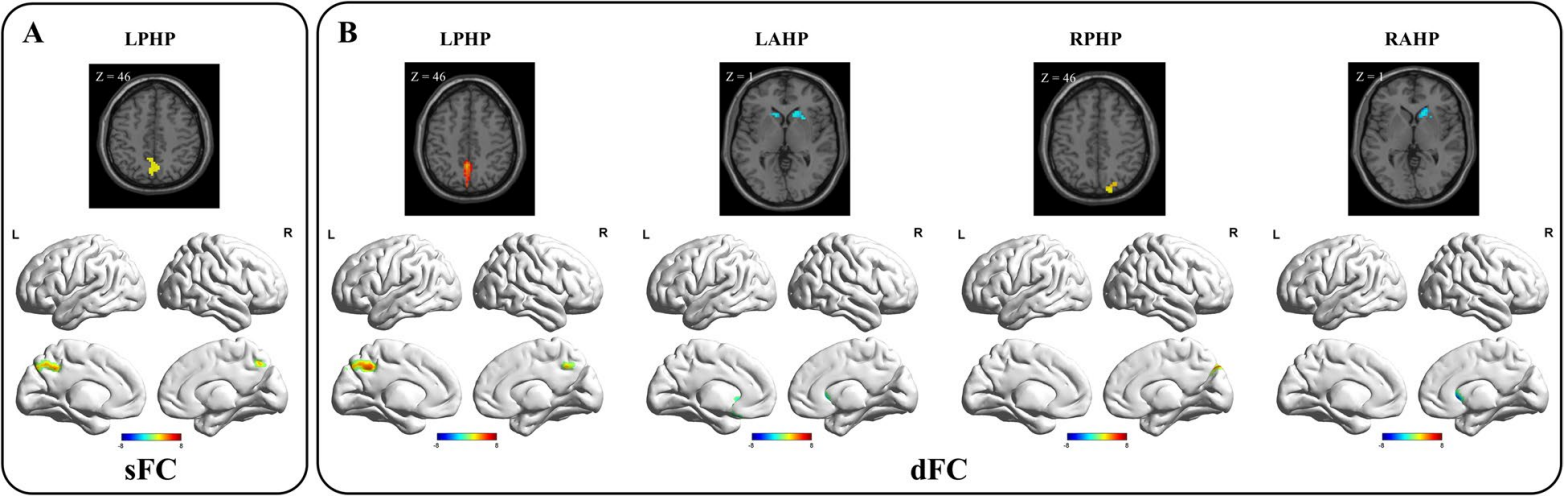
Differences in anterior-posterior hippocampal sFC and dFC between the SCD and HC groups. **A** In the two independent sample *t* test of sFC, the SCD group exhibited decreased sFC values between the left posterior hippocampus and precuneus compared with the HC group. **B** In the two independent sample *t* test of dFC, the SCD group exhibited increased dFC variability between the bilateral posterior hippocampus and the bilateral precuneus, decreased dFC variability between the bilateral anterior hippocampus and the bilateral caudate nucleus, and decreased dFC variability between the left anterior hippocampus and the left olfactory cortex compared with the HC group. LAHP, left anterior hippocampus; LPHP, left posterior hippocampus; RAHP, right anterior hippocampus; RPHP, right posterior hippocampus; HC, healthy controls; SCD, subjective cognitive decline

**Fig. 3:**
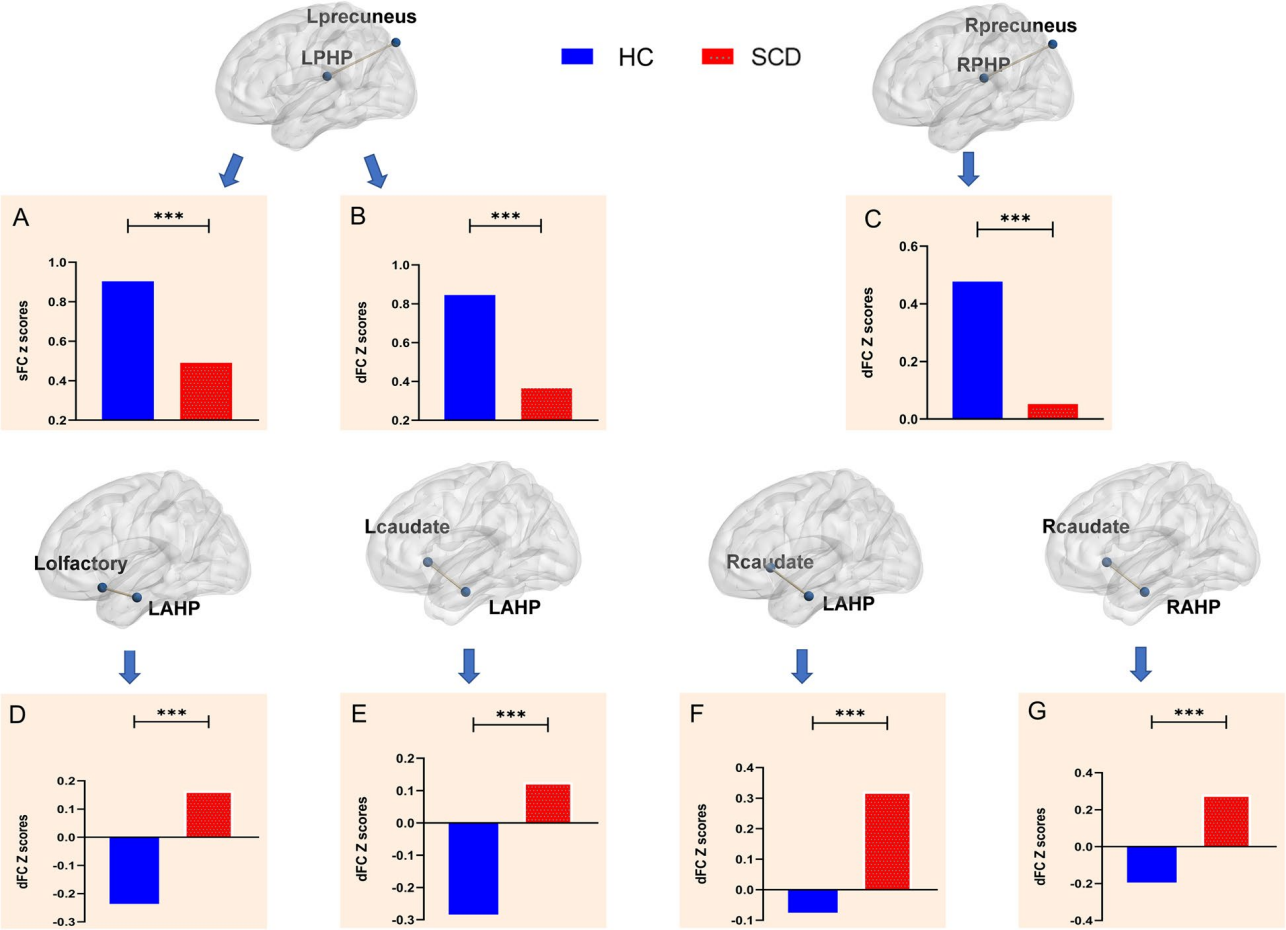
Comparison of anterior-posterior hippocampal sFC and dFC values between the SCD and HC groups. Compared with the HC group, the SCD group exhibited: **A** decreased sFC between the left posterior hippocampus and the left precuneus; **B** decreased dFC variability between the left posterior hippocampus and the left precuneus; **C** decreased dFC variability between the right posterior hippocampus and the right precuneus; **D** increased dFC variability between the left anterior hippocampus and the left olfactory cortex; **E** increased dFC variability between the left anterior hippocampus and the left caudate nucleus; **F** increased dFC variability between the left anterior hippocampus and the right caudate nucleus; and **G** increased dFC variability between the right anterior hippocampus and the right caudate nucleus. ∗Statistically significant at the 0.05 level (2-tailed); ∗∗Statistically significant at the 0.01 level (2-tailed); ∗∗∗Statistically significant at the 0.001 level (2-tailed). LAHP, left anterior hippocampus; LPHP, left posterior hippocampus; RAHP, right anterior hippocampus; RPHP, right posterior hippocampus; HC, healthy controls; SCD, subjective cognitive decline

In the two independent samples *t* test of sFC, the SCD group exhibited decreased sFC values between the left posterior hippocampus and precuneus compared with the HC group. In the two independent samples *t* test of dFC variability, the SCD group exhibited decreased dFC variability between the bilateral posterior hippocampus and bilateral precuneus, increased dFC variability between the bilateral anterior hippocampus and bilateral caudate nucleus, and decreased dFC variability between the left anterior hippocampus and left olfactory cortex compared with the HC group (Table 2 and Figs. 2 and 3).

**Table 2:** Comparison of functional connectivity between the HC and SCD groups

Comparison	Brain regions	Peak MNI			Cluster size	*F*
		*X*	*y*	*z*		
**sFC**						
**Left posterior hippocampus**						
HC > SCD	Left precuneus	− 3	− 75	42	78	3.93
**dFC**						
**Left posterior hippocampus**						
HC > SCD	Left precuneus	− 3	− 51	42	103	4.94
**Left anterior hippocampus**						
HC < SCD	Left olfactory cortex	− 6	18	− 15	15	− 3.60
	Left caudate nucleus	− 15	21	− 6	17	− 3.49
	Right caudate nucleus	15	21	0	29	− 3.56
**Right posterior hippocampus**						
HC > SCD	Right precuneus	15	− 84	45	44	4.85
**Right anterior hippocampus**						
HC < SCD	Right caudate nucleus	15	21	0	55	− 5.40

*Abbreviations*: *HC* healthy controls, *SCD* subjective cognitive decline, *sFC* static functional connectivity, *dFC* dynamic functional connectivity. Gaussian random field (GRF) corrected (voxel *p* < 0.001, cluster *p* < 0.05)

### Relationships between altered functional connectivity and neuropsychological scale scores

**Fig. 4:**
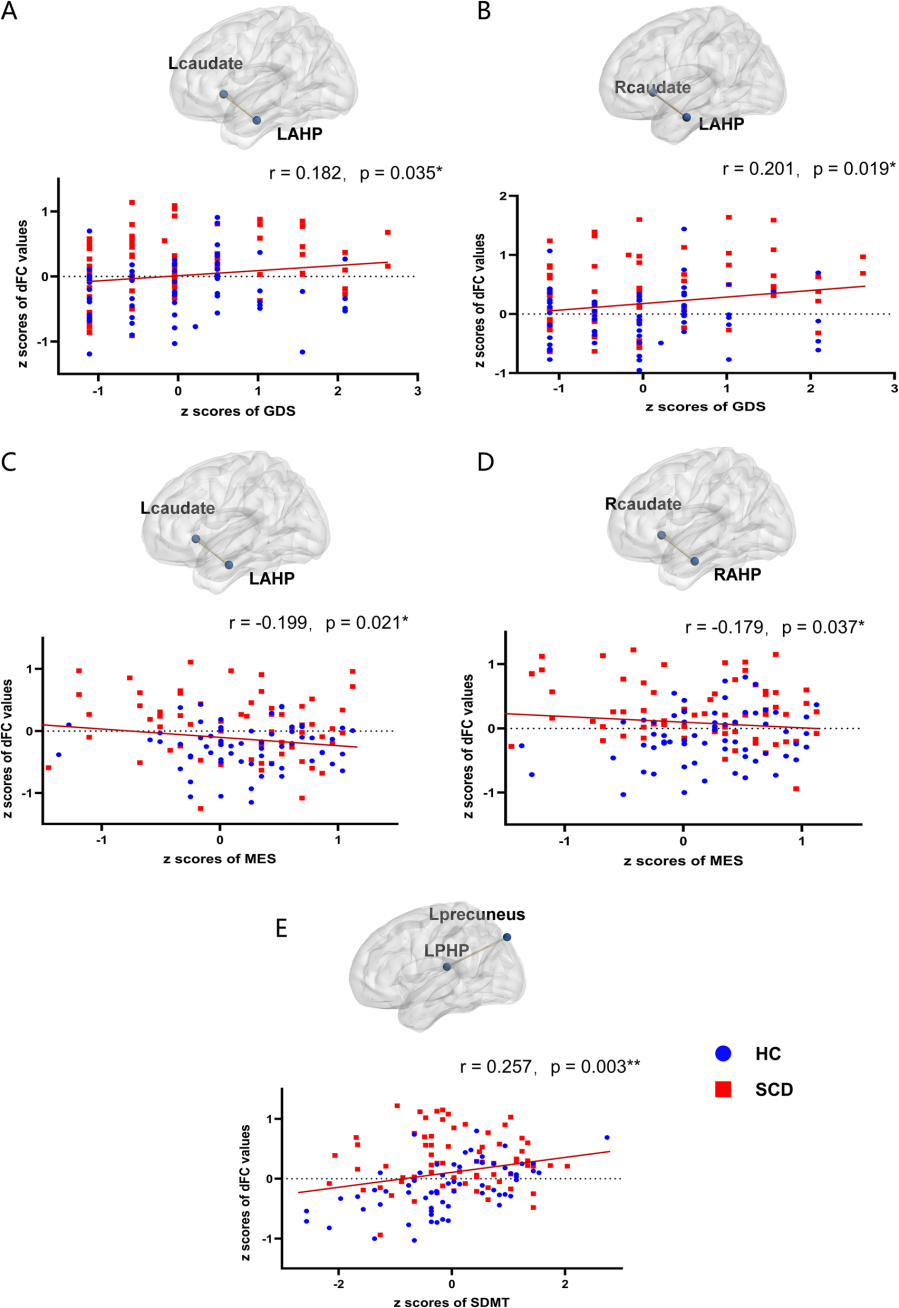
The correlation between abnormal functional connectivity values and neuropsychological variables. **A** The correlation between GDS and dFC between the left anterior hippocampus and the left caudate nucleus (*r* = 0.182, *p* = 0.035). **B** The correlation between GDS and dFC between the right anterior hippocampus and the right caudate nucleus (*r* = 0.201, *p* = 0.019). **C** The correlation between MES and dFC between the left anterior hippocampus and the left caudate nucleus (*r* = 0.199, *p* = 0.021). **D** The correlation between MES and dFC between the right anterior hippocampus and the right caudate nucleus (*r* = 0.179, *p* = 0.037). **E** The correlation between SDMT and dFC between the left anterior hippocampus and left precuneus (*r* = 0.257, *p* = 0.003). ∗Statistically significant at the 0.05 level (2-tailed); ∗∗Statistically significant at the 0.01 level (2-tailed); ∗∗Statistically significant at the 0.001 level (2-tailed). LAHP, left anterior hippocampus; LPHP, left posterior hippocampus; RAHP, right anterior hippocampus; RPHP, right posterior hippocampus; HC, healthy controls; SCD, subjective cognitive decline; MES, memory and executive screening; SDMT, Symbol-Digit Modality Test; GDS, Geriatric Depression Scale

Significant associations between altered FC and neuropsychological scale scores are summarized in Fig. 4. In the whole sample, the *z* scores of GDS were positively correlated with dFC variability between the left anterior hippocampus and left caudate nucleus (*r* = 0.182, *p* = 0.035) (Fig. 4A) and between the left anterior hippocampus and right caudate nucleus (*r* = 0.201, *p* = 0.019) (Fig. 4B). The *z* scores of the MES were negatively correlated with dFC between the left anterior hippocampus and the left caudate nucleus (*r* = 0.199, *p* = 0.021) (Fig. 4C) and dFC between the right anterior hippocampus and the right caudate nucleus (*r* = 0.179, *p* = 0.037) (Fig. 4D). The *z* scores of SDMT were positively correlated with dFC between the left posterior hippocampus and left precuneus (*r* = 0.257, *p* = 0.003) (Fig. 4E). There was no significant correlation between the bilateral posterior hippocampus and other brain regions. There was no significant correlation between abnormal sFC values and neuropsychological scale scores (*p* > 0.05).

## Discussion

This study is the first to investigate anterior-posterior hippocampal functional abnormalities in subjects with SCD by using a seed-based sFC and dFC approach. Compared to healthy controls, subjects with SCD showed (1) decreased sFC between the left posterior hippocampus and left precuneus, (2) decreased dFC variability between the bilateral posterior hippocampus and precuneus, increased dFC variability between the bilateral anterior hippocampus and caudate nucleus, and increased dFC variability between the left anterior hippocampus and left olfactory cortex. Additionally, the attention scores were positively correlated with the dFC variability between the left posterior hippocampus and left precuneus, and the dFC variability between the bilateral anterior hippocampus and caudate nucleus was positively correlated with depression scores and negatively correlated with global cognition scores.

Previous studies have demonstrated that meta-state activation of dynamic brain networks is associated with AD progression [42], and Aβ deposition is positively associated with dynamic but not static FC in preclinical AD [43]. Moreover, recent analyses showed altered dFC variability within the triple networks (the default mode network, the salience network and the executive control network) with MCI [44] and altered intrinsic brain activity with SCD [45]. Based on previous studies, the present study selected the bilateral anterior-posterior hippocampus as seeds and found that the altered anterior-posterior hippocampal dFC variability is already present with SCD using the sliding-window dynamic method. On the one hand, the present study confirms previous findings that subjects with SCD show decreased sFC between the left posterior hippocampus and left precuneus [11, 13]. On the other hand, subjects with SCD showed both abnormal brain mean FC (sFC) and abnormal brain functional stability and variability in the time dimension (dFC), and the abnormal dFC was more widespread than the sFC. Therefore, we believe that dFC is a powerful supplement to sFC [46], and dFC may more sensitively reflect abnormal connectivity [47]. Overall, a combination of sFC and dFC may provide a new perspective for exploring the brain pathophysiological mechanisms in SCD.

The present analyses of rs-fMRI suggested that cognitive function is positively correlated with dFC between the left posterior hippocampus and the left precuneus and negatively correlated with dFC between the bilateral anterior hippocampus and caudate nucleus. Thus, both the posterior and anterior hippocampus may be involved in cognitive processing. Studies investigating cognitive function have identified two major cortical networks that are highly connected with the hippocampus—the anterior-temporal and the posterior-medial systems [48]. Recent studies have shown that tau-PET binding in the anterior hippocampus is directly related to memory function [49], early Aβ deposition occurs in the posterior-medial system, such as the posterior cingulate cortex and the precuneus, in preclinical AD [50], and altered dFC between the hippocampus and amygdala is consistent with high pathological tau deposition [51]. Furthermore, the strength of connections between the right caudate nucleus and the hippocampus showed a correlation in memory performance [52]. Therefore, our study confirmed that pathological changes precede the appearance of clinical symptoms in the AD disease spectrum because SCD exhibits intact cognitive function but abnormal FC [53]. Thus, we speculate that the findings of higher dFC values between the posterior hippocampus and the precuneus may indicate a compensatory mechanism for the decreased function of the anterior hippocampus and caudate nucleus for cognitive function in SCD individuals. It is necessary to conduct a longitudinal neuroimaging study to further explore the relationship between hippocampal dFC, AD pathology, and the development of dementia.

Previous studies have also shown that individuals with SCD may have mild subclinical depressive symptoms, which increases the risk of progression to objective cognitive impairment [26]. Interestingly, the current study also showed that depressive symptoms were positively correlated with the dFC of the anterior hippocampus and caudate nucleus of the hippocampus. The hippocampus is the core brain area of emotional response [54], especially the anterior hippocampus [55]. Our previous studies have also found that patients with late-life depression exhibit structural and functional abnormalities in the hippocampus [56]. Additionally, longitudinal research showed that regional Aβ load in the hippocampus and bilateral caudate nucleus are associated with the progression of depression during a 3-year follow-up [57]. Therefore, the present results suggest that modulating the dFC of the anterior hippocampus may contribute to alleviating depressive symptoms, and therapeutic interventions for depressive symptoms may alleviate the psychological burden of negative emotions in people with SCD.

The present research also found increased dFC between the left anterior hippocampus and the left olfactory cortex in SCD individuals, which is a powerful supplement to previous studies showing that SCD individuals exhibit significant cortical thinning in the olfactory cortex compared with healthy controls [58]. It is well known that AD pathology begins in the transentorhinal region and then moves to the entorhinal cortex before affecting the hippocampus [59, 60]. Additionally, Aβ and tau preferentially deposit in the anterolateral entorhinal cortex, and stronger connectivity is associated with increased tau deposition [61, 62]. Our previous study also demonstrated that odor identification dysfunction is already present with SCD and deepens with disease severity in the AD spectrum [63]. However, the relationship between olfactory dysfunction, connectivity of the hippocampus with olfactory regions and the risk of dementia in SCD individuals requires further exploration.

### Limitations

The present study has several limitations. First, we were unable to draw a causal relationship between FC and cognitive function/affection due to the cross-sectional design of this study, and longitudinal studies are underway to elucidate the role of sFC/dFC in the whole AD spectrum. Second, the present study did not include information on the biomarkers of AD (such as Aβ and tau) for subjects with SCD. Thus, we could not determine the relationship between the rs-fMRI indicators and AD pathology. Third, the current study did not include MCI or AD, and it remains unclear whether the above indicators of rs-fMRI will change with the development of the AD spectrum. Fourth, we adopted the widely used 50 TRs and 30 TRs sliding-window length approach to extract FC dynamics in the current study. To avoid potential bias, future studies can consider using other window lengths or fresh extraction methods, such as the point-process method [64].

## Conclusions

SCD individuals exhibited abnormal sFC and dFC variability in the anterior-posterior hippocampus. Abnormal dFC in the posterior hippocampal system is associated with subtle changes in cognitive function, and changed dFC in the anterior hippocampal system is associated with subtle changes in emotion and cognition. The dFC may be more sensitive than the sFC to reflect the functional abnormalities of the hippocampus in SCD individuals, and the combination of sFC and dFC provides a new perspective for exploring the brain pathophysiological mechanisms in SCD and offers potential neuroimaging biomarkers for the early diagnosis and intervention of AD.

## Supplementary Information

**Additional file 1: Supplementary material 1.** Results of 50 TRs.

**Additional file 2: Supplementary material 2.** Results obtained without smoothing.

## Data Availability

The data, which support this study, is not publically available but may be provided upon reasonable request.

## Abbreviations

AD: Alzheimer’s disease
AVFT: Animal verbal fluency test
AVLT-DR: Auditory verbal learning test delay recall
Aβ: β-amyloid
BOLD: Blood oxygen level-dependent
dFC: Dynamic functional connectivity
DMN: Default mode network
EPI: Echo-planar imaging
FC: Functional connectivity
fMRI: Functional magnetic resonance imaging
FOV: Field of view
GDS: Geriatric depression scale
GRF: Gaussian random field
HC: Healthy controls
MCI: Mild cognitive impairment
MES: Memory and executive screening
MMSE: Mini-mental state examination
MNI: Montreal Neurological Institute
MRI: Magnetic resonance imaging
PET: Positron emission tomography
ROCF: Rey-Osterrieth complex figure test
ROIs: Regions of interest
r-s fMRI: Rest static functional magnetic resonance imaging
SCD: Subjective cognitive decline
SDMT: Symbol digit modalities test
sFC: Static functional connectivity
TDA: Temporal Dynamic Analysis
TE: Echo time
TMTB: Trail-making test
TR: Repetition time
