# Supplementary material for: Static and dynamic functional connectivity variability of the anterior-posterior hippocampus with subjective cognitive decline
Source: Alzheimers Res Ther. 2022 Sep 3;14:122. doi: 10.1186/s13195-022-01066-9 (PMC9440588; doi:10.1186/s13195-022-01066-9)
Supplement: Supplementary file 1 — Additional file 1: Supplementary material 1. Results of 50 TRs. [file 13195_2022_1066_MOESM1_ESM.docx]

**
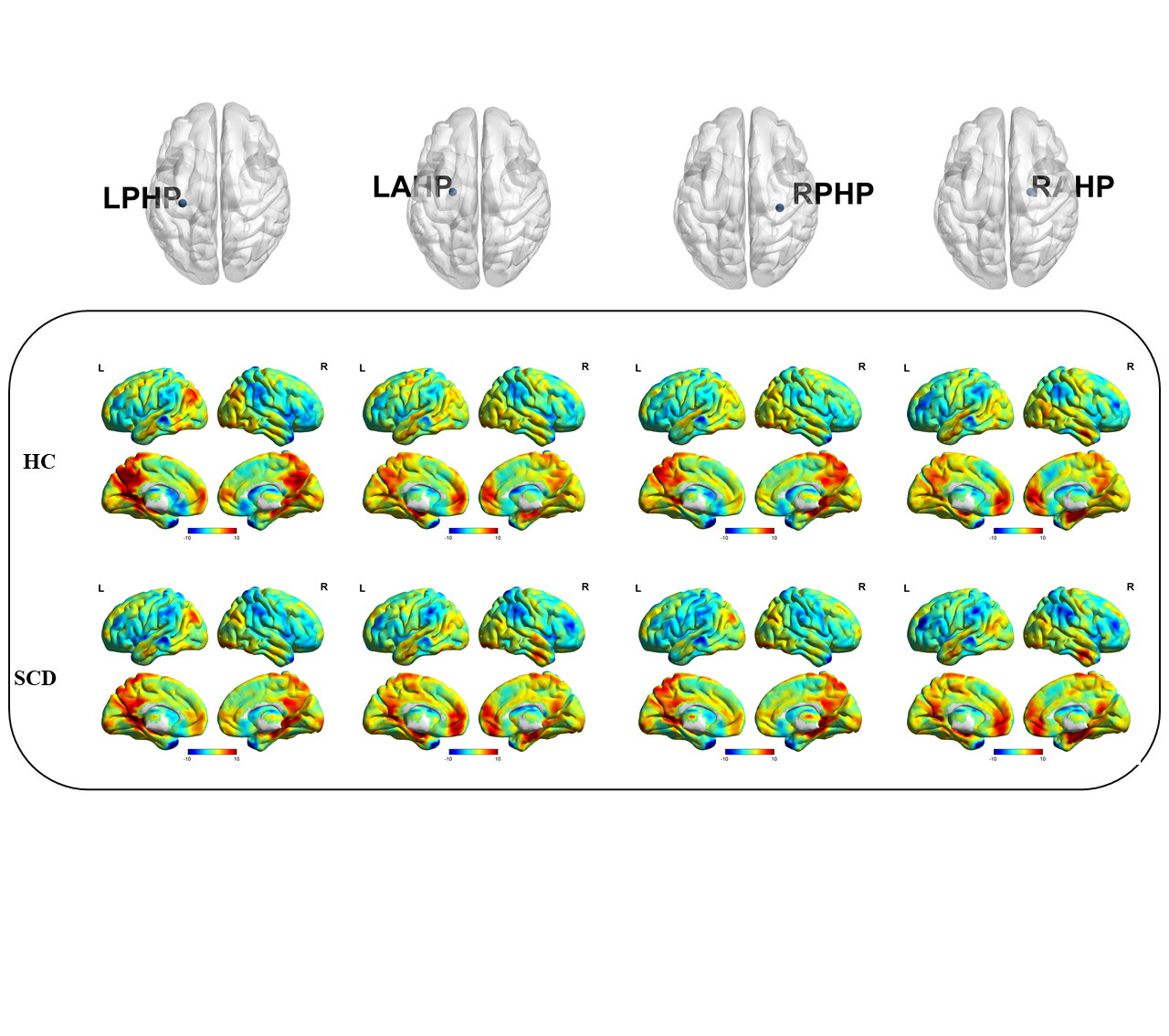
**

**Figure 1.** The one-sample t-test of dFC (30TRs-60 sec) patterns of bilateral anterior and posterior hippocampus in SCD and HC group. *The one-sample t-test of dFC (30TRs-60 sec) showed that bilateral posterior hippocampus and anterior hippocampus with high dFC values mainly connected to bilateral frontal cortex, temporal cortex, and parietal cortex.*

*Abbreviations: LAHP: left anterior hippocampus, LPHP: left posterior hippocampus, RAHP: right anterior hippocampus, RPHP: right posterior hippocampus. HC, healthy controls; SCD: subjective cognitive decline*

**Table 1**. Comparison of dFC (30TRs-60 sec) between HC and SCD group.

| Comparison | Brain Regions | Peak MNI | | | | Cluster size | F |
| --- | --- | --- | --- | --- | --- | --- | --- |
|  |  | X | y | | z |  |  |
| **Left posterior hippocampus** | | | | | | | |
| HC > SCD | Left precuneus | 0 | -51 | 42 | | 87 | 4.53 |
| HC < SCD | Right insula | 36 | 9 | 12 | | 67 | -4.75 |
| **Left anterior hippocampus** | | | | | | | |
| HC < SCD | Left caudate nucleus and olfactory cortex | -15 | 21 | -3 | | 80 | -3.92 |
| **Right** **posterior hippocampus** | | | | | | | |
| HC > SCD | Right precuneus | 15 | -84 | 45 | | 33 | 5.24 |
| **Right anterior hippocampus** | | | | | | | |
| HC < SCD | Right caudate nucleus | 15 | 21 | 0 | | 52 | -4.91 |

*Abbreviations: HC, healthy controls; SCD: subjective cognitive decline; dFC: dynamic functional connectivity. Gaussian random field (GRF) corrected (voxel p < 0.001, cluster p < 0.05).*


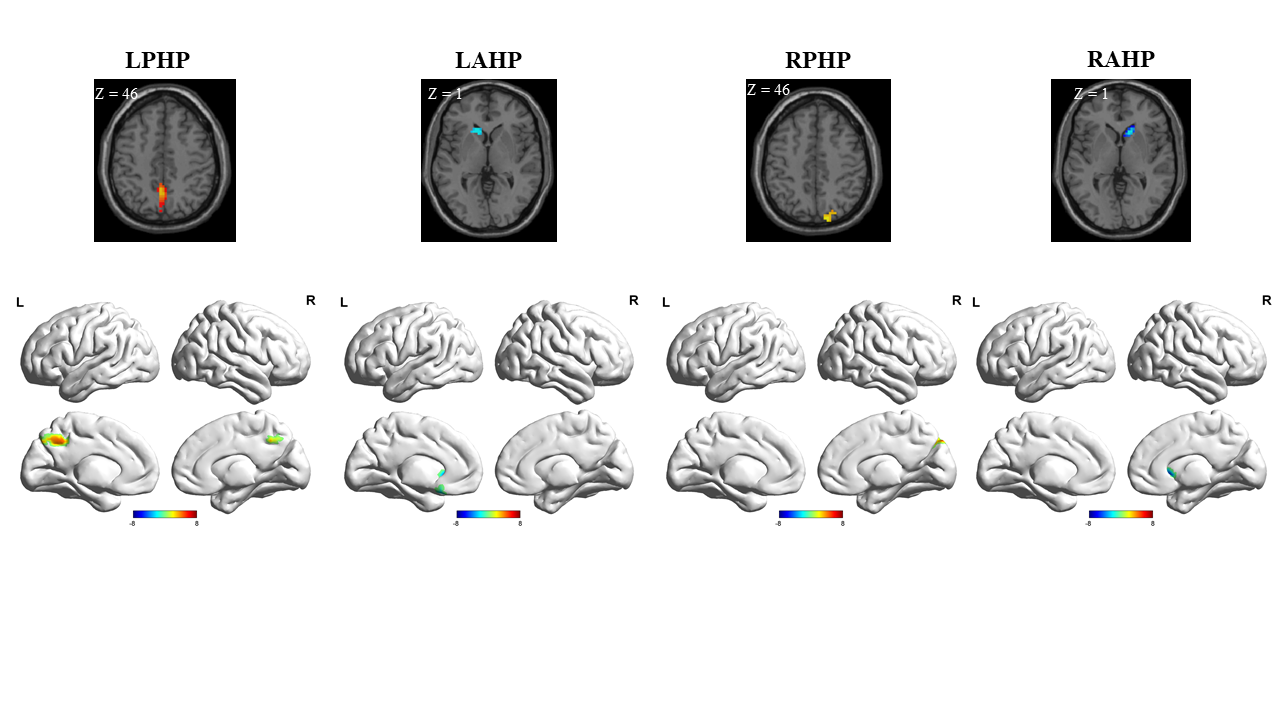


**Figure 2.** Difference of anterior-posterior hippocampal dFC (30TRs-60 sec) between SCD and HC group. *In the two independent samples t test of dFC, compared with the HC group, the SCD group exhibited increased dFC variability between the bilateral posterior hippocampus and the bilateral precuneus, and decreased dFC variability between the bilateral anterior hippocampus and the bilateral caudate nucleus, and decreased dFC variability between the left anterior hippocampus and the left olfactory cortex.*

*Abbreviations: LAHP: left anterior hippocampus, LPHP: left posterior hippocampus, RAHP: right anterior hippocampus, RPHP: right posterior hippocampus. HC, healthy controls; SCD: subjective cognitive decline*


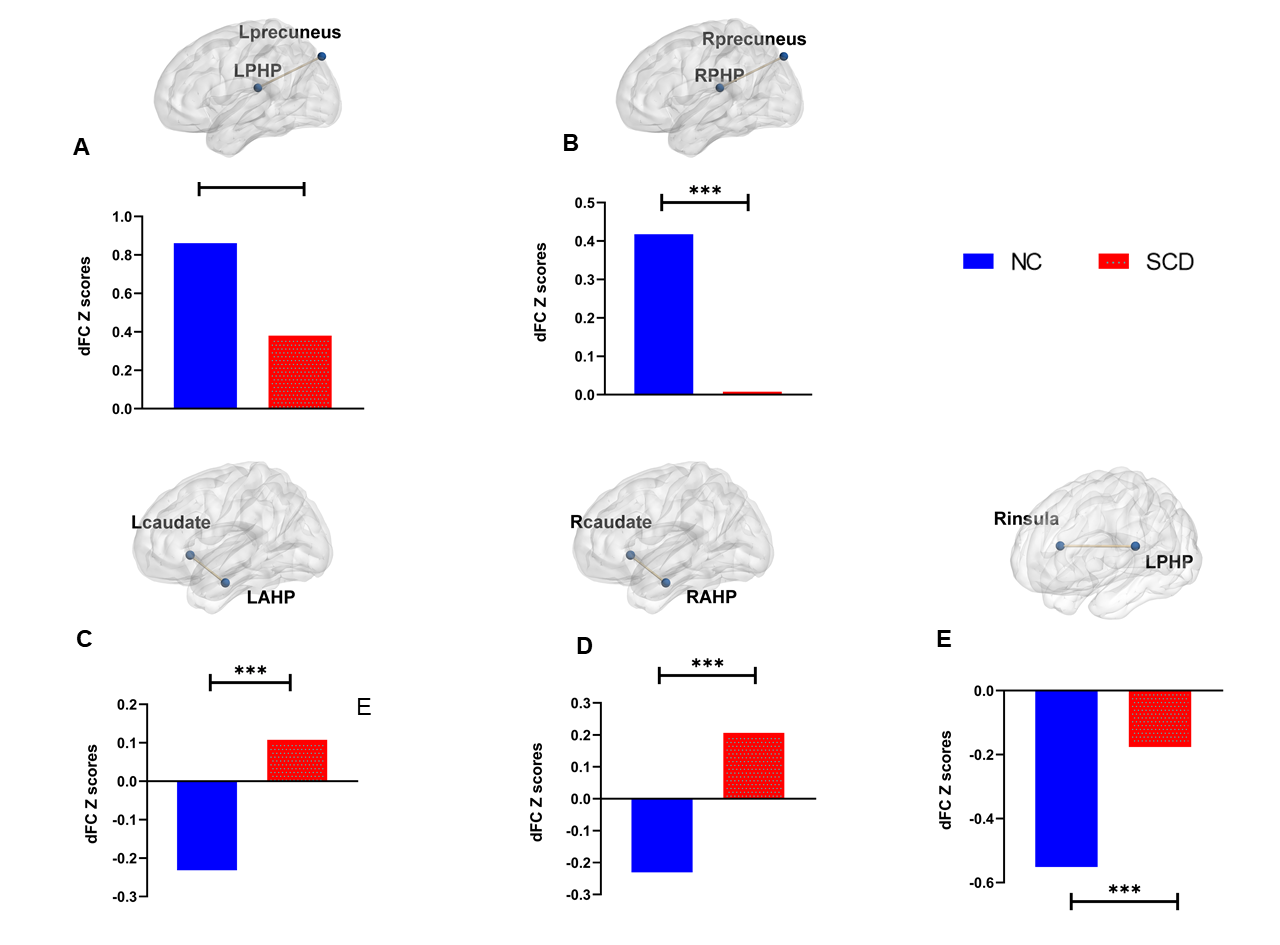


**Figure 3.** Comparison of anterior-posterior hippocampal dFC values between SCD and HC group. *Compared with the HC group, the SCD group exhibited A) decreased dFC variability between the left posterior hippocampus and the left precuneus, B) decreased dFC variability between the right posterior hippocampus and the right precuneus; C) increased dFC variability between the left anterior hippocampus and the left caudate nucleus and olfactory cortex; D) increased dFC variability between the right anterior hippocampus and the right caudate nucleus; E) increased dFC variability between the left posterior hippocampus and the right insula.*

*∗Statistically significant at the 0.05 level (2-tailed); ∗∗Statistically significant at the 0.01 level (2-tailed); ∗∗∗Statistically significant at the 0.001 level (2-tailed).*
